# Diminished muscle integrity in patients with fibrodysplasia ossificans progressiva assessed with at-home electrical impedance myography

**DOI:** 10.1038/s41598-022-25610-7

**Published:** 2022-12-03

**Authors:** Alexander Farid, Emma Golden, Sara Robicheau, Alice Hu, Kin Cheung, Paul B. Yu, Seward B. Rutkove, Jaymin Upadhyay

**Affiliations:** 1 Department of Anesthesiology, Critical Care and Pain Medicine, Boston Children’s Hospital, Harvard Medical School, Boston, MA 02115 USA; 2 Myolex Inc, Boston, MA USA; 3 BioSAS Consulting, Inc., Wellesley, MA USA; 4 Division of Cardiology, Cardiovascular Research Center, Massachusetts General Hospital, Harvard Medical School, Boston, MA USA; 5 Department of Neurology, Beth Israel Deaconess Medical Center, Boston, MA USA; 6 Department of Psychiatry, McLean Hospital, Harvard Medical School, Belmont, MA, USA

**Keywords:** Diseases, Endocrine system and metabolic diseases, Rheumatic diseases, Diagnostic markers, Prognostic markers, Paediatric research

## Abstract

Fibrodysplasia ossificans progressiva (FOP) is an ultra-rare disorder involving skeletal dysplasia and heterotopic ossification (HO) of muscle and connective tissue. We aimed to define a novel biomarker in FOP that enables reliable assessment of musculoskeletal tissue integrity. Considering logistical difficulties that FOP patients often face, our goal was to identify an at-home biomarker technique. Electrical impedance myography (EIM) is a non-invasive, portable method that can inform on muscle health. 15 FOP patients (age 10–52) and 13 healthy controls were assessed. Using EIM, multiple muscle groups were characterized per participant in a 45-min period. The Cumulative Analogue Joint Involvement Scale (CAJIS) was implemented to determine mobility burden severity. We additionally evaluated physical activity levels via a Patient-Reported Outcomes Measurement Information System (PROMIS)-based questionnaire. Relative to controls, FOP patients demonstrated significantly lower regional and whole-body phase values at 50 kHz and 100 kHz, indicating more diseased muscle tissue. Lower whole-body phase and reactance values, and higher resistance values, were associated with greater FOP burden (CAJIS score range: 4–30) and lower physical activity levels at 50 kHz and 100 kHz. This study points to the potential utility of EIM as a clinical biomarker tool capable of characterizing muscle integrity in FOP.

## Introduction

Fibrodysplasia ossificans progressiva (FOP; OMIM#135100) is an ultra-rare (1:800,000–1:3,000,000 prevalence worldwide) inherited condition that arises from missense mutations of the type I bone morphogenetic protein (BMP) receptor, activin A receptor type 1 (*ACVR1*)^1–3^. The mutant form of ACVR1 mediates aberrant activin A-dependent osteogenic signaling, facilitating heterotopic ossification (HO) of skeletal muscles, tendons, ligaments, and fascia, and subsequent loss of mobility, pain, and reduced quality-of-life^4^. Preclinical and clinical observations have shown that induction or growth of HO may be preceded by painful, edematous soft tissue swellings termed flare-ups^5–7^. There is also a likelihood that sub-clinical, pathological events occur during what is otherwise considered a ‘quiescent’ period, where ongoing processes and symptoms may occur outside of detectable flare-ups^8–10^.

Historically, imaging techniques such as X-rays, computed tomography (CT), ^99m^TC-MDP Single Photon Emission Computed Tomography (SPECT), ^18^F-NaF positron emission tomography (PET), and magnetic resonance imaging (MRI) have been utilized to detect FOP lesions in musculoskeletal tissue as well as monitor disease progression or burden^8,11–17^. Although imaging markers may be useful for clinical management and for characterizing FOP pathophysiology, there are limitations associated with these approaches. For example, with nuclear medicine-based measures (e.g., ^99m^TC-MDP or ^18^F-NaF-PET/CT), radiation exposure may limit the frequency of use, particularly in pediatric FOP populations. For some FOP patients, undergoing MRI may also be difficult considering the small-bore size of the magnet in conjunction with physical constraints that patients with FOP often harbor. Moreover, soft tissue edema on MRI and ^18^F-NaF-PET/CT activity are respectively associated with localized tissue inflammation and osteogenic activity typically associated with flares, but do not always correlate with the clinical course or predict sites of future HO^15^. To complement clinical and imaging data, recent studies have sought to identify disease and flare-up-associated biomarkers in plasma samples derived from patients with FOP^18^, which if validated in subsequent work, may provide a novel means to classify disease stages of FOP or further the understanding of mechanistic pathways implicated in FOP.

The composition and structural integrity of skeletal muscle is also altered in FOP. Disturbances in muscle integrity associated with the presence of HO, edema, and degeneration alters histological and consequently biophysical properties of muscle. Therefore, we evaluated electrical impedance myography (EIM), a non-invasive technology in which a painless electrical current at a range of frequencies is passed through muscle and resulting voltages at the skin surface are measured^19,20^. Importantly, EIM equipment is portable, and measurements can be completed on a large number of muscle groups with the patient positioned comfortably in under 45 min.

EIM has been used clinically in multiple rare musculoskeletal and neuromuscular conditions^21–24^. EIM has not been applied and validated in FOP populations. The objective of the current study was to determine whether the bioelectric properties as measured by EIM in pediatric and adult patients with FOP were altered relative to matched healthy controls. We hypothesize that FOP patients with further progressed disease will exhibit greater alterations in bioelectric properties in individual muscle groups and at the whole-body level, defined as the averaged EIM-based metric across all muscle groups evaluated in each study participant.

Additionally, this investigation was conducted during the COVID-19 pandemic at which time travel, particularly for high risk patient populations, was inadvisable and many FOP patients or families were rightfully reluctant to come to a hospital for study participation. In order to accommodate all study participants, we adapted EIM to an entirely at-home and virtual platform.

## Materials and methods

This investigation was approved by the Boston Children’s Hospital (BCH) Institutional Review Board (IRB). All research was performed in accordance with relevant guidelines/regulations and performed in accordance with the Declaration of Helsinki. Eligible study participants provided informed consent using an IRB-approved virtual process. For individuals below 18 years of age, assent was provided and informed consent was obtained from their parent or legal guardian.

### Study participants

Fifteen individuals diagnosed with FOP (7 males; 8 females; age range: 10–52 years) and 13 healthy controls (7 males; 6 females; age range: 11–51 years) were evaluated in this study. FOP patients were recruited via FOP-specific forums organized by the International FOP Association (IFOPA). Additional recruitment was aided by clinicians currently treating FOP patients. Healthy age- and gender-matched controls were recruited from the Boston metropolitan area. Enrolled FOP patients had a confirmed, classic gain-of-function R206H mutation in *ACVR1*^25^. Based on known regions of HO, the current FOP patient population spanned a spectrum of disease burden (See also patient-specific Cumulative Analogue Joint Involvement Scale (CAJIS) scores)^26^. No patient was in an active flare-up state during the study. Healthy control subjects did not report any chronic illnesses or experienced any recent or ongoing musculoskeletal injury. Prior to EIM data acquisition, participants provided or confirmed demographic information, height, weight, physical activity, and whole-body (0–10 scale) pain levels. Physical activity (short-form) and pain levels were evaluated using questionnaires derived from the Patient-Reported Outcomes Measurement Information System (PROMIS; http://www.healthmeasures.net) database. Questionnaires were administered and completed online using REDCap (https://www.project-redcap.org).

### EIM data acquisition

**Figure 1:**
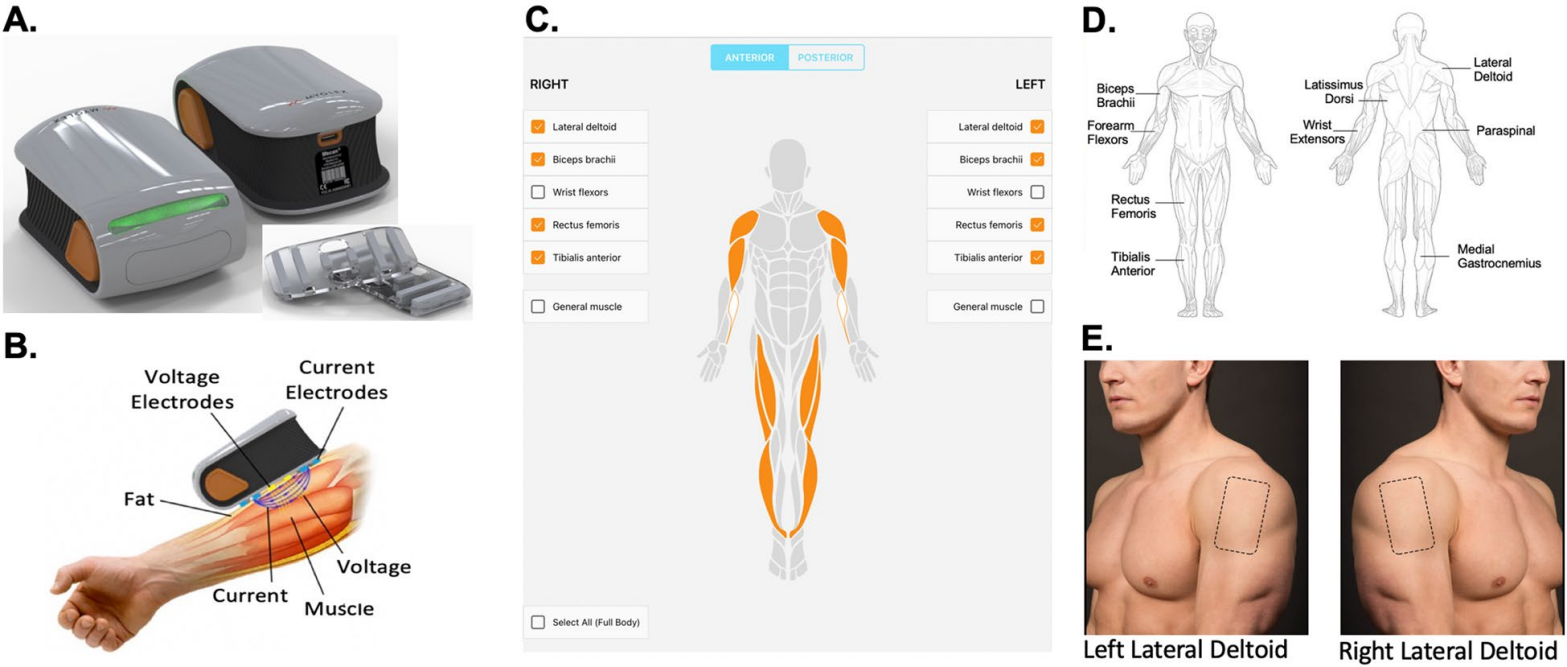
EIM experimental setup. (**A**) Handheld mScan EIM device, which is connected via Bluetooth to an iPad. EIM sensors are shown on the bottom right. The mScan data acquisition app is also installed on the iPad. An additional iPad is used for video conferencing enabling study staff to monitor data acquisition and the participants in real time. (**B**) The EIM device sends electrical impulses from the skin towards deeper muscle tissue. The integrity of underlying muscle tissue will impact the propagation of electrical impulses between electrodes. (**C**) Front page of the mScan app as displayed on the iPad and viewable by the individual acquiring the EIM data as well as the study staff remotely connected to the iPad via Zoom. (**D**) In this study, multiple anterior and posterior muscle groups were evaluated in each study participant. (**E**) Instruction provided on the mScan app notifying the individual acquiring the data where to place the EIM device on the FOP patient or healthy control subject. Example is shown for the left and right lateral deltoid. Similar instructions and images were displayed for other muscle groups evaluated during the virtual study.

An EIM device (mScan; Myolex Inc) along with an accompanying iPad (Apple Inc.) with EIM data acquisition software and a BCH-approved Zoom account were shipped to each study participant (Fig. 1). The EIM device, which is placed on the surface of the skin, consists of electrodes that are made from a hydrophilic foam that is infused with an electrolyte solution. Each current electrode is 2.5 cm from the voltage electrode and the two voltage electrodes are 1.75 cm apart. The EIM device is calibrated rigorously against capacitor/resistor networks. The EIM device is accurate to within ± 1% of the expected impedance values between 10 and 1000 kHz.

**Figure 2:**
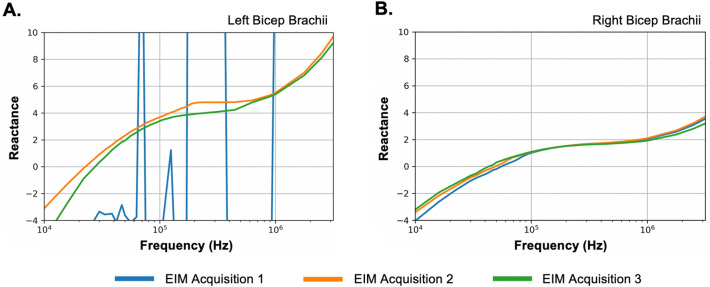
Real-time QA/QC of EIM data. (**A**) EIM traces from the left bicep brachii from FOP 003. In one EIM acquisition (blue trace) shows large spikes caused by accidental patient movement or movement of the EIM device by the care provider, warranting a rescan of the left bicep brachii. Subsequent scans of the left bicep brachii (green and orange traces) showed no artifact and were incorporated in data analysis. (**B**) Raw EIM traces from FOP 003 from the right bicep brachii. The EIM traces were collected during same study session and showed no signal artifact.

Prior to the virtual EIM study session, study participants were asked to have clean skin surfaces and avoid use of any lotions or oils. At the start of the virtual study visit, one individual, such as a primary caregiver, parent, or significant other was trained to use the data collection software and acclimated to the handheld EIM device. The data acquisition software provides a depiction of each anatomical location the EIM device should be place on. Furthermore, study staff visually verified via Zoom video that the primary caregiver, parent, or significant other placed the EIM device on the correct anatomical location prior data collection. Two to three test runs on a single muscle region (e.g., forearm) were carried out to (i) verify acquisition of robust EIM signals void of any artifacts, (ii) further help the trainee and study participant comprehend the data collection process and (iii) provide the trainee with further guidance where and when needed by the study staff. As part of the implemented QA/QC procedure, EIM waveforms were viewed in real-time during the training session as well as the subsequent acquisition period (Fig. 2). Each training session lasted for 10–15 min.

Following this training session, EIM measurements were obtained from multiple muscle groups bilaterally. The upper extremity muscle groups included the lateral deltoid, biceps brachii, wrist extensors, forearm flexors. Lower extremity muscle groups included rectus femoris, tibialis anterior, and medial gastrocnemius. Axial muscle groups included the latissimus dorsi and paraspinal muscles. During EIM data acquisition, study participants were asked to remain in a relaxed and comfortable position. Contact impedance was reduced by thoroughly moistening the skin with saline (via separate application of saline via a wipe) and letting the skin remain consistently moistened for 3–5 min before EIM measurements were taken. This procedure helped to ensure effective hydration of the stratum corneum. Following this procedure, the EIM device was placed on the specific anatomical region being examined and prompted by the mScan app, and, as during training, was confirmed by the study staff member in real-time using video conferencing. Measurements on each muscle group were repeated two to three times, depending on scan quality. Muscle specific data was reviewed in real-time by study staff, allowing for quick confirmation of high quality EIM traces. The most common EIM signal artifact (i.e., spike in EIM trace) was caused by trainee or subject movement, in which case the scan at a specific muscle was repeated. Following confirmation of data quality, the trainee and study participant were automatically prompted to acquire data on the next muscle group. EIM data collection from each muscle group was completed in a ~ 60 s period. For some patients with FOP with very extensive disease and who utilized specialized ambulation equipment, paraspinal muscles were not assessed with EIM.

### CAJIS

Following the EIM portion of the virtual study visit, each FOP patient was assessed using the CAJIS^26^, a clinical tool informing on whole-body and regional functional mobility burden in patients with FOP. Functional or movement-related limitations were defined for multiple musculoskeletal joints, where mobility was determined as (i) normal or < 10% deficit (score of 0), (ii) 10–90% deficit (score of 1), or (iii) ≥ 90% deficit or functionally ankylosed (score of 2). Additional accounts of mobility (i.e., ability to walk) or need for assistance with daily activities are also considered in CAJIS, where the total score ranges from 0 to 30.

### Data analysis

EIM Data Preprocessing: Each unilateral muscle group and each EIM trace (EIM scan frequency range: 1–10,000 kHz) underwent further QA/QC to identify any signal distortions or artifacts that may have been missed during real-time data assessment. Any EIM trace with signal artifact was discarded and not included in subsequent data analysis. For each muscle, the average EIM trace was generated and utilized for further EIM analysis. Bioelectrical properties at 50 kHz and 100 kHz were determined based on previous studies showing that these frequencies have high sensitivity to detect differences between diseased and muscle tissue and for having high reproducibility^19,20,27^. At 50 kHz and 100 kHz, the capacitive nature of the muscle is most apparent and thus provides the strongest muscle signature. For each muscle group, resistance (*R*, a measure of difficulty passing EIM current through the tissue), reactance (*X*, a measure of the capacitive effects of the cell membranes) and the phase (*θ*, equal to the arctan [reactance/resistance]) was quantified at 50 kHz and 100 kHz. The primary EIM biomarker endpoint was defined as the *θ* at 50 kHz. To quantify regional EIM-based measures, defined as upper left, upper right, lower left, and lower right regions, data for the following muscle groups were averaged as follows; (i) upper region—lateral deltoid, biceps brachii, wrist extensors, forearm flexors, latissimus dorsi and paraspinal muscles and (ii) lower region—rectus femoris, tibialis anterior, and medial gastrocnemius.

### Statistical analysis

The Shapiro–Wilk test was implemented to test for normality of the data. Some data met the normality assumption but not all. To be conservative, we did not make any assumptions about the distribution of the data, and as a result the Wilcoxon's two-sample test was implemented. To determine significant differences between patients with FOP and healthy controls, the Wilcoxon two-sample test was performed. Effect sizes (ES) for all measures of interest were also calculated (ES: mean of healthy controls—mean of FOP patients)/Pooled STD; Pooled STD = SQRT[(std1^2^ + std2^2^)/2], where std1 and std2 are the standard deviation of healthy control and FOP patients, respectively. To determine whether and how EIM base measures associated with clinical benchmark measures (i.e., CAJIS scores), the Pearson correlation coefficient was calculated.

## Results

### Study participant characteristics

Study participant demographics, height, weight, body mass index, physical activity, and pain levels are described in Table 1. However, as expected, physical activity and pain levels were lower and higher, respectively, in FOP patients relative to controls. The current FOP patient cohort consisted of individuals who required assisted ambulation, which can contribute to low physical activity levels. Furthermore, higher self-reported pain levels by patients are in accord with prior work noting the prevalence of pain as a prevalent symptom of FOP^9,10,28^.

**Table 1:** Demographics and baseline characteristics.

Patient	Age (y)	Gender	Height (cm)	Weight (kg)	BMI (kg/m^2^)	Pain score	Physical activity	Total CAJIS score
FOP01	11	F	149.9	31.3	13.9	0	42.4	5
FOP02	9	F	139.7	24	12.3	7	28.8	15
FOP03	44	M	180.3	65.8	20.2	3	28.8	26
FOP04	15	M	154.9	37.6	15.7	4	39.2	7
FOP05	11	M	132	33	18.9	0	47.8	11
FOP06	52	F	147	60	27.8	2	28.8	24
FOP07	33	F	160	64	25.0	7	28.8	30
FOP08	11	M	122	23	15.5	5	40.4	11
FOP09	39	F	175	125	40.8	6	28.8	11
FOP10	29	F	168	88	31.2	3	32.6	18
FOP11	34	M	185	72	21.0	6	32.6	4
FOP12	25	F	168	95	33.7	6	28.8	14
FOP13	42	M	178	52	16.4	0	28.8	25
FOP14	33	F	163	68	25.6	1	34.5	19
FOP15	52	M	181	82	25.0	4	34.5	24
Mean (SD)	29.5 (14.99)	–	160 (21)	55.6 (23.44)	21.8 (6.43)	3.5 (2.56)	33.71 (6.10)	16.3 (8.21)
HC01	11	M	150	41	18.2	0	47.8	–
HC02	14	M	173	56	18.7	0	53.3	–
HC03	11	F	155	40	16.6	0	60.8	–
HC04	32	F	157	56	22.7	0	49.6	–
HC05	10	M	147	33	15.3	1	62.1	–
HC06	23	M	186	82	23.7	0	54.3	–
HC07	12	F	163	45	16.9	0	59.5	–
HC08	51	M	170	69	23.9	1	71.7	–
HC09	41	M	175	76	24.8	0	71.7	–
HC10	39	F	165	58	21.3	0	39.2	–
HC11	23	F	172	55	18.6	0	37.9	–
HC12	31	M	185	70	20.5	0	42.4	–
HC13	50	F	151	60	26.3	4	41.4	–
Mean (SD)	26.8 (15.03)	–	170 (13)	57.0 (14.66)	20.6 (3.49)	0.4 (0.87)	53.20 (11.51)	–

*BMI* body mass index, *SD* standard deviation.

Pain score—0–10 scale; Average Pain Intensity over prior 7 days.

PROMIS Physical Activity Scale (Short Form)—Raw scores converted to T-score for each patient; Higher scores correspond to more physical activity.

Cumulative Analogue Joint Involvement Scale: CAJIS—total score ranging from 0 to 30.

### Altered whole-body and regional bioelectric properties

**Figure 3:**
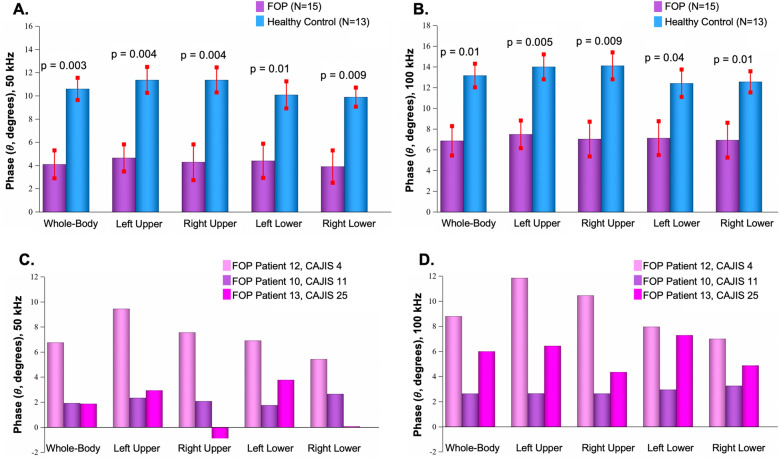
Whole-body and regional phase in patients with FOP and healthy controls. The mean ± standard error (SE) phase (*θ*) values for whole-body and regional (left upper, right upper, left lower, and right lower) assessments are compared between FOP and healthy control cohorts at 50 kHz (**A**) and 100 kHz (**B**). At both frequencies as well as for whole-body or regional assessments, mean θ values were consistently lower in the FOP cohort relative to healthy control population. P-values (two-tailed) are reported from Wilcoxon two-sample test. Effect size range for 50 kHz: 1.13–1.58. Effect size range for 100 kHz: 0.95–1.36. Group-level findings for reactance (*X*) and resistance (*R*) values are provided in Table 2. The mean *θ* values are reported at 50 kHz (**C**) and 100 kHz (**D**) at whole body and regional (left upper, right upper, left lower, and right lower) levels for three male FOP patients with varying CAJIS scores. Across muscle groups, *θ* was consistently higher in FOP patient 12 (CAJIS score 4) when compared to those with higher CAJIS scores. At 50 kHz in particular, EIM was able to differentiate between more severely afflicted muscle regions, as indicated by lower *θ*, and regions with healthier unaffected muscle. This suggests that EIM is capable of detecting regional changes in muscle tissue integrity in FOP.

Each EIM-based endpoint (*θ*, *X*, and *R*) was assessed as a whole body, composite measure as well as on a regional basis. Separate assessments were made at 50 kHz and 100 kHz. Whole-body and regional *θ* at 50 kHz and 100 kHz was significantly reduced in patients with FOP relative to healthy controls (Fig. 3A,B). Whole-body and regional variability of *θ* at 50 kHz and 100 kHz at the single patient-level could also be observed. Although significant, the reduction in *θ* at 100 kHz for the FOP cohort was slightly less robust as compared to 50 kHz data. *X* at 50 kHz and 100 kHz were also significantly reduced in FOP patients compared to healthy controls, while *R* was marginally higher in patients with FOP at either frequency (Table 2). Collectively, inter-group differences in whole-body and regional *θ*, *X*, and *R* demonstrate the ability of EIM to detect altered bioelectric properties in patients with FOP. While large effect sizes were observed for several EIM-based measures, *θ* and *X* at 50 kHz at the whole-body as well as the regional level, showed particularly high sensitivity for differentiating patients with FOP from control subjects. Additionally, these results provide support for virtual EIM data acquisition.

**Table 2:** Comparison of reactance and resistance values at 50 and 100 kHz in patients with FOP versus healthy controls.

Parameter	Body part	Statistics	FOP (N = 15)	Healthy controls (N = 13)
Reactance 50 kHz	Whole body	Mean (SD)	1.8 (1.62)	3.8 (0.66)
		Median	1.5	4.1
		P-value	0.0052	
		Effect size	1.62	
	Left upper	Mean (SD)	2.3 (1.78)	4.5 (0.72)
		Median	2.2	4.4
		P-value	0.0016	
		Effect size	1.63	
	Right upper	Mean (SD)	2.0 (2.44)	4.3 (0.67)
		Median	2.0	4.6
		P-value	0.0023	
		Effect size	1.33	
	Left lower	Mean (SD)	1.8 (1.90)	3.4 (1.04)
		Median	1.7	3.6
		P-value	0.0238	
		Effect size	1.06	
	Right lower	Mean (SD)	1.4 (1.78)	3.3 (0.74)
		Median	1.2	3.1
		P-value	0.0091	
		Effect size	1.38	
Reactance 100 kHz	Whole body	Mean (SD)	2.8 (1.47)	4.2 (0.62)
		Median	2.1	4.4
		P-value	0.0082	
		Effect size	1.24	
	Left upper	Mean (SD)	3.3 (1.65)	4.8 (0.59)
		Median	3.0	4.8
		P-value	0.0091	
		Effect size	1.22	
	Right upper	Mean (SD)	2.9 (2.14)	4.7 (0.66)
		Median	2.5	4.8
		P-value	0.0141	
		Effect size	1.13	
	Left lower	Mean (SD)	2.7 (1.60)	3.7 (0.85)
		Median	3.0	3.9
		P-value	0.0697	
		Effect size	0.78	
	Right lower	Mean (SD)	2.4 (1.60)	3.6 (0.63)
		Median	1.9	3.7
		P-value	0.0323	
		Effect size	1.05	
Resistance 50 kHz	Whole body	Mean (SD)	31.4 (11.20)	23.5 (7.35)
		Median	26.2	22.1
		P-value	0.0527	
		Effect size	− 0.84	
	Left upper	Mean (SD)	33.4 (11.60)	25.8 (9.70)
		median	31.1	24.0
		P-value	0.0697	
		Effect size	− 0.71	
	Right upper	Mean (SD)	32.7 (12.73)	24.5 (7.65)
		Median	24.5	22.2
		P-value	0.0914	
		Effect size	− 0.79	
	Left lower	Mean (SD)	30.8 (10.88)	21.6 (6.18)
		Median	27.3	21.1
		P-value	0.0264	
		Effect size	− 1.04	
	Right lower	Mean (SD)	28.3 (10.59)	21.1 (6.35)
		Median	24.8	19.5
		P-value	0.0636	
		Effect size	− 0.82	
Resistance 100 kHz	Whole body	Mean (SD)	30.0 (11.63)	21.0 (7.62)
		Median	24.6	19.7
		P-value	0.0394	
		Effect size	− 0.91	
	Left upper	Mean (SD)	31.8 (11.82)	23.0 (9.97)
		Median	30.2	20.6
		P-value	0.0323	
		Effect size	− 0.81	
	Right upper	Mean (SD)	31.2 (13.15)	21.8 (7.89)
		Median	24.3	19.9
		P-value	0.0527	
		Effect size	− 0.86	
	Left lower	Mean (SD)	29.5 (11.56)	19.5 (6.52)
		Median	26.6	19.8
		P-value	0.0264	
		Effect size	− 1.07	
	Right lower	Mean (SD)	27.0 (11.11)	18.8 (6.58)
		Median	23.6	16.2
		P-value	0.0357	
		Effect size	− 0.90	

*SD* standard deviation.

P-value derived from a Wilcoxon two-sample test comparing the mean difference between FOP patients and healthy controls.

Effect Size (Mean of Healthy Control − mean of FOP Patients)/Pooled STD; Pooled STD = sqrt[(std1^2^ + std2^2^)/2] std1 and std2 are the standard deviation of Healthy Control and FOP, respectively.

### Associations among whole-body and regional EIM measures with CAJIS and baseline characteristics

In Fig. 3C,D, *θ* values are shown for three adult male FOP patients with varying-levels of functional mobility burden (i.e., CAJIS scores of 4, 11, and 25). For FOP patients with less disease burden (e.g., FOP patient 12), higher *θ* values were quantified in anatomical regions less affected by the disease. From single-subject data, *θ* values at 50 kHz were more closely associated with CAJIS scores compared to *θ* values at 100 kHz.

**Figure 4:**
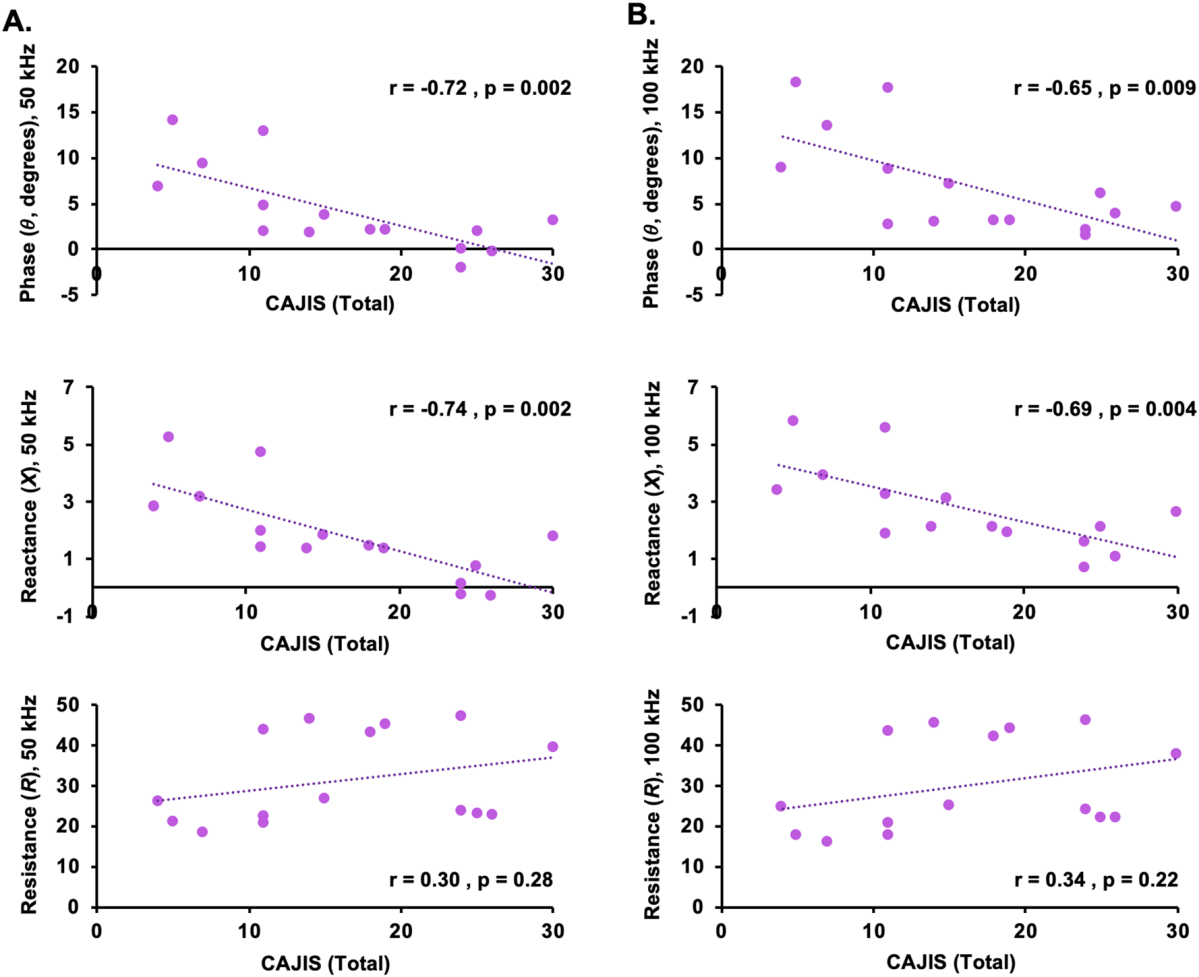
Association between total CAJIS scores and EIM measures (*θ*, *X*, and *R*). At 50 kHz (**A**) and 100 kHz (**B**), robust negative correlations between CAJIS scores and *θ* (top row) as well as CAJIS and *X* were observed. A non-significant positive correlation trend between CAJIS scores and R (bottom row) was present. Correlation results in general were comparable between 50 and 100 kHz. Pearson’s correlation analysis results and two-tailed p-values are reported. Whole-body or composite EIM measures were solely utilized in correlation analysis. Negative correlations identified for *θ* and *X* combined with group-level comparisons shown in Fig. 3 indicate more diseased muscle tissue in patients with FOP.

**Figure 5:**
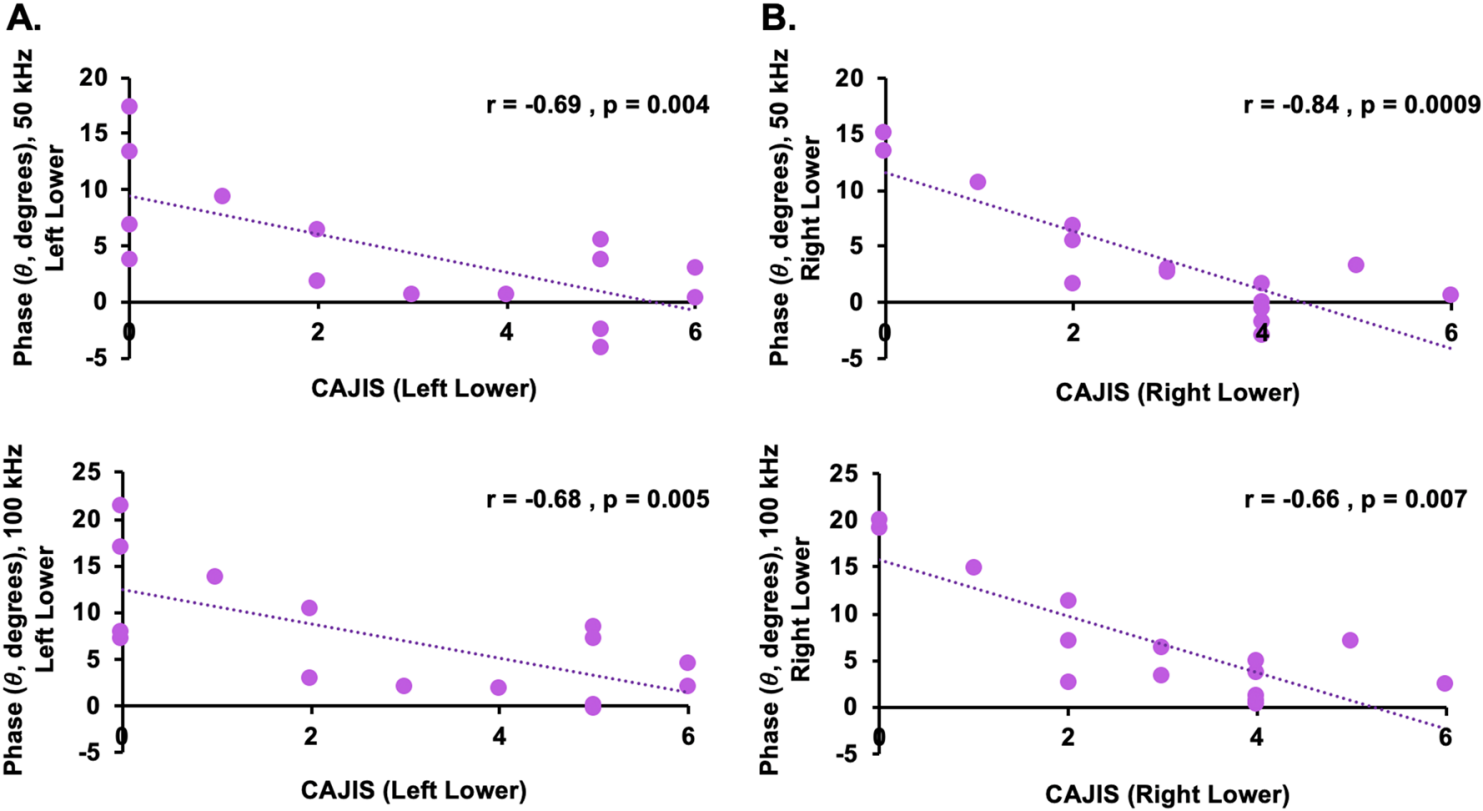
Association between regional CAJIS scores and *θ*. At both 50 and 100 kHz, robust negative correlations between CAJIS scores and *θ* in the left lower (**A**) and right lower (**B**) regions were observed. Such localized associations further support the ability of EIM to detect both regional and whole-body changes in muscle tissue integrity in FOP patients.

At 50 kHz (Fig. 4A) and 100 kHz (Fig. 4B), lower whole-body *θ* and *X* significantly correlated with higher composite CAJIS scores. A non-significant trend of higher *R* and higher CAJIS scores was present. To demonstrate the presence of regional association between EIM and regional functional mobility deficits, the relation between left lower body *θ* at both 50 kHz (Fig. 5A) and 100 kHz (Fig. 5B) with corresponding left lower body CAJIS scores were assessed. Similar to whole-body findings, lower regional *θ* at 50 or 100 kHz significantly associated with lower regional CAJIS scores. Moreover, in accord with earlier studies, CAJIS scores significantly correlated with the age of FOP patients (r = 0.65; p = 0.009). In FOP patients, age was negatively associated with *θ* at 50 kHz (r = −0.77; p = 0.0008) and *θ* at 100 kHz (r = −0.75; p = 0.001). In healthy controls, a correlation between age and *θ* at 50 kHz (r = −0.26; p = 0.39) and *θ* at 100 kHz (r = −0.37; p = 0.21) was not present. Higher whole-body *θ* as well as *X* values were significantly correlated with higher levels of physical activity (Table 3). Conversely, higher physical activity scores corresponded to lower whole-body *R* values. These trends were present at both the 50 kHz and 100 kHz frequencies and in the FOP population and healthy control cohort. The presence of significant correlations between EIM measures and CAJIS in addition to physical activity levels suggest clinical and behavioral bases for altered *θ*, *X*, and *R* values in the FOP cohort. A chronic state of immobilization is also likely to occur in conjunction with structural changes in muscle fibers, which in turn would contribute to altered bioelectric properties measured with EIM (see Discussion). Finally, significant correlation between EIM measures (*θ*, *X*, and *R*) and other patient characteristics such as whole-body pain levels were not present. Thus, clinical pain is an unlikely clinical symptom that contributes to altered EIM properties in FOP patients.

**Table 3:** Correlation analysis, whole-body EIM versus physical activity.

Parameter	Body part	FOP (N = 15)	Healthy controls (N = 13)
Phase (θ) 50 kHz	Whole body	r = 0.64, p = 0.010	r = 0.61, p = 0.028
Phase (θ) 100 kHz	Whole body	r = 0.68, p = 0.0050	r = 0.55, p = 0.051
Reactance (*X*) 50 kHz	Whole body	r = 0.61, p = 0.016	r = 0.21, p = 0.49
Reactance (*X*) 100 kHz	Whole body	r = 0.63, p = 0.011	r = 0.40, p = 0.18
Resistance (*R*) 50 kHz	Whole body	r = − 0.53, p = 0.041	r = − 0.59, p = 0.035
Resistance (*R*) 50 kHz	Whole body	r = − 0.56, p = 0.030	r = − 0.58, p = 0.036

Pearson’s correlation coefficients (r) and two-tailed p-values are reported.

## Discussion

The current results demonstrate that EIM is an efficient and accessible method capable of detecting aberrant bioelectric properties of muscle tissue in patients with FOP. Whole-body and regional EIM endpoints (*θ* and *X*) at 50 kHz and 100 kHz were found to be lower in patients with FOP compared to healthy subjects. Equally interesting was the observation that EIM data at the single-patient level indicated regional variability, where some muscle groups of patients with FOP fell within a normal range for *θ* (see left upper for FOP Patient 12, Fig. 2) and other muscles were more affected as indicated by lower *θ* values. The heterogeneity in EIM measures, whether from an inter- or intra-patient perspective, suggests that EIM may be sensitive to anatomically localized pathological processes (e.g., regional HO growth). However, factors such as widespread HO within a FOP patient may underpin less variability in EIM data.

The pathobiological basis for the current set of EIM findings (i.e., changes in whole-body and regional *θ*, *X*, and *R* values) in patients with FOP is likely multifactorial. We hypothesize that myopathic processes and perhaps relatedly, inflammatory mechanisms, present at a regional or systemic level would ultimately alter the conduction of electrical currents^29,30^. More specifically, lower *X* values may be attributed to changes in cell membranes or muscle atrophy as damaged cell membranes yield a decrease in tissue capacitance. The presence of extensive HO may contribute to the low *X* values as bone is a poor conductor as a result of low levels of fluid or electrolytes. Additionally, *R*, a measure of difficulty for electrical currents passing through tissue, was in contrast elevated in the FOP cohort. This finding was in accord with the current understanding that HO or calcification in soft tissue would ultimately increase the resistance for a passing electrical current. Thus, the lower *θ* values observed in patients with FOP are hypothesized to reflect a combination of myofiber structural alterations and HO. A focus for future EIM studies in FOP patients would be a determination of how *θ*, *X*, or *R* regionally vary during a flare-up, while a finer dissection of pathophysiological mechanisms of FOP underpinning changes in EIM properties warrants further investigation and perhaps is best suited using preclinical models of FOP^5^.

We also sought to evaluate whether and how EIM parameters relate to a benchmark functional measure of FOP, CAJIS^26^. Here, strong negative correlations were found between CAJIS scores and *θ* and *X* at both 50 and 100 kHz, on a whole-body and regional scale (i.e., left-lower). As a progressive condition, FOP disease burden, as indicated by more extensive HO and more severe loss of functional mobility as captured by CAJIS, frequently worsens with age. In line with this trend, EIM-based endpoints also significantly associated well with age among our FOP patient population, a finding not replicated in healthy control subjects. To further evaluate the ability of EIM to reflect patients’ functional status, we utilized a PROMIS-based questionnaire to evaluate physical activity level. We found positive correlation between *θ* and *X* values with level of physical activity, while *R* was negatively correlated with this same measure. As our other analyses have demonstrated, *R* is negatively correlated with disease affliction. With more extensive disease, as expected, PROMIS data indicated decreased engagement in physical activity. In turn, the reported correlations between EIM endpoints and physical activity level are consistent with correlations between EIM endpoints and disease affliction.

The negative association between EIM endpoints and CAJIS scores suggest that abnormal EIM metrics reflect muscle dysfunction secondary to chronic immobilization, yet the possibility that aberrant bioelectric properties of muscle as a causal factor precipitating HO cannot be ruled out without further investigation. Nonetheless, these data point to the utility of EIM as a biomarker method to objectively evaluate a FOP patient’s functional status and possibly after further investigation, a tool to monitor disease over time.

Prior to the current investigation, EIM has been established as a flexible tool to assess muscle in healthy subjects as well as neuromuscular and musculoskeletal conditions^21–24,29–32^. Yet, the implementation of EIM in a virtual setting is unique to this study. Importantly, despite virtual data collection by an individual other than a study staff member, we observed that healthy control subject EIM data are well within the range of what has been reported for *θ*, *X*, and *R* at 50 and 100 kHz. For instance, in separate studies, the mean *θ* at 50 kHz across multiple muscle groups in healthy male children and healthy adults was approximately ~ 11° degrees and ~ 14°, respectively^31,32^. In this study’s virtual healthy control cohort involving pediatric and adult participants, the mean *θ* at 50 kHz across multiple muscle groups was similarly ~ 11°. Inter-study differences in mean *θ* values may have risen from variability in in age, gender, and BMI distribution as well as measures such as physical activity levels. Nonetheless, the alignment and consistency between in-person and virtual EIM evaluations provides support for using EIM in a virtual manner, whether it is in healthy controls or in patients with FOP.

There are a number of challenges associated with identifying much needed novel and sensitive biomarkers in FOP. Aside from patient availability, given the ultra-rare status of FOP, logistical hurdles and contraindications towards employing normally routine procedures (i.e., biopsy or MRI) can further limit biomarker development in this condition. Imaging techniques such as PET, SPECT, CT, or X-Ray can provide critical information on FOP disease activity and burden, yet the frequency of implementation may be limited, particularly in the pediatric FOP population. Nonetheless, validation of non-invasive and objective biomarkers capable of monitoring musculoskeletal health in FOP would be advantageous in clinical and research settings alike. Thus, the current study aimed to test the utility of EIM in FOP, and determined that EIM-based endpoints that inform on muscle health, particularly *θ* and *X* at 50 kHz, can differentiate between patients with FOP and matched, healthy controls. These measures also significantly associate with CAJIS, a functional measure of mobility validated in FOP populations^26^, as well as with reported physical activity levels, another measure of functionality. Moreover, the COVID-19 pandemic imposed further limitations and hurdles in performing clinical research in patients with FOP. Ultimately, the pandemic drove a creative adaptation (i.e., transitioning to a virtual platform) of an already-flexible tool in EIM, and allowed for execution of an at-home clinical biomarker research study, which likely has implications for future studies carried out beyond the COVID-19 pandemic.

By adapting the in-person EIM study visit for a virtual platform, a number of insights have been garnered. Firstly, study participants and the individual who collected EIM data have consistently reported that appropriate operation and handling of the EIM device was easy to learn. FOP patients and families have similarly expressed how valuable they feel at-home modalities that inform on their disease status or overall health can be given the logistical difficulties with travel many individuals face. Relatedly and from a research perspective, moving our acquisition paradigm to a virtual platform has significantly decreased the burden on our participants. Additionally, a virtual scanning modality allows for wider reach, which is particularly important given the ultra-rare status of FOP. From a clinical standpoint for FOP, the painful flare-ups that often precede an episode of HO induction and growth are difficult to predict. Patients have repeatedly emphasized the need to accurately detect flare-up states and thus potential HO lesions. While further research is necessary to determine the ability of EIM to detect the underlying soft tissue changes associated with a flare-up, the findings of this study serve as a good foundation that demonstrates the ability of EIM to detect disease-related changes in FOP in general.

There are several limitations in this study that warrant discussion. First, our study analyzed muscle groups, which were in large part evaluated in prior EIM investigations. On one hand, this approach provided guidance in terms of the expected range for EIM values, which was important considering the novel use of EIM in patients with FOP as well as in a virtual study setting. Yet, limiting the muscle groups evaluated also led to the exclusion of certain anatomical regions where HO lesions notably form in FOP (e.g., neck, upper back or jaw). Examination with EIM of small muscle groups such as those in the facial area with current EIM equipment is challenging. Second, in patients with widespread presence of HO and who use specialized wheelchairs, paraspinal muscles were particularly difficult to access with the EIM device, yet all other muscle groups were evaluated. Third, this study was cross-sectional and can only inform on the value of EIM as a tool to assess an acute state of muscle health in FOP patients. We propose that future work in FOP populations should incorporate EIM within a multidisciplinary clinical research framework that monitors patients longitudinally. Such study designs are critical in order to determine how EIM measures track with other benchmark measures over time, as well as to ascertain the value of EIM as a method that can identify acute versus chronic changes, predict disease progression, and relatedly, detect episodic events such as HO expansion or flare-ups. Integrating non-contrast MRI or MR spectroscopy, when possible to employ in a FOP patient, may provide important insights into how altered muscle tissue properties may relate to EIM-based measures. Fourth, as no FOP patient was in a confirmed flare-up state, the utility of EIM to detect and monitor flare-ups could not be established. Finally, we can only speculate about the possible pathophysiological mechanisms driving abnormal EIM measurements in FOP patients. Localized or systemic myopathy, chronic inflammation, atrophy, HO, prolonged immobilization or combination of factors could modulate the bioelectric properties of muscle tissue. An opportunity for further research may entail evaluation of EIM with other objective and more established methods such as imaging (^18^F-NaF PET/CT, CT, X-Ray, and MRI) or quantification of circulating markers^18,33–35^.

In conclusion, EIM has tremendous potential as an objective, non-invasive, and convenient clinical research tool that can be utilized to detect diseased muscle in pediatric and adult patients with FOP. Given EIM’s inherit flexibility and ease of operation, this method can be incorporated as a standalone measure or in combination with current approaches standardized in clinical or research settings. The results presented herein, provide a necessary foundation for further determining the utility of EIM as a biomarker method that can be used to track disease progression, monitor patients in flare-up states, and, possibly inform on treatment effects in FOP.

## Disclosures

SR and AU are employee of Myolex Inc. KC is the founder of BioSAS Consulting. SBR has equity in and serves a consultant and scientific advisor to Myolex, Inc., and Haystack Diagnostics, Inc., companies that design impedance devices for clinical and research use; he is also a member of the Myolex's Board of Directors. The companies also have an option to license patented impedance technology of which SBR is named as an inventor. PBY is a co-founder of Keros Therapeutics, which develops therapeutics for hematological and musculoskeletal diseases which target TGF-ß signaling pathways. SBR is compensated for work on the company’s scientific advisory board and owns equity in the publicly-traded company. SR, AU, and SBR were not involved in the acquisition of data during the course of this study, but did provide technical support. All other authors have nothing to disclose. All authors approved the manuscript.

## Data Availability

The data that support the findings of this study are available from the corresponding author upon reasonable request.
